# GenExp: An Interactive Web-Based Genomic DAS Client with Client-Side Data Rendering

**DOI:** 10.1371/journal.pone.0021270

**Published:** 2011-07-05

**Authors:** Bernat Gel Moreno, Xavier Messeguer Peypoch

**Affiliations:** Software Department, UPC-BarcelonaTech, Barcelona, Spain; University of Toronto, Canada

## Abstract

**Background:**

The Distributed Annotation System (DAS) offers a standard protocol for sharing and integrating annotations on biological sequences. There are more than 1000 DAS sources available and the number is steadily increasing. Clients are an essential part of the DAS system and integrate data from several independent sources in order to create a useful representation to the user. While web-based DAS clients exist, most of them do not have direct interaction capabilities such as dragging and zooming with the mouse.

**Results:**

Here we present GenExp, a web based and fully interactive visual DAS client. GenExp is a genome oriented DAS client capable of creating informative representations of genomic data zooming out from base level to complete chromosomes. It proposes a novel approach to genomic data rendering and uses the latest HTML5 web technologies to create the data representation inside the client browser. Thanks to client-side rendering most position changes do not need a network request to the server and so responses to zooming and panning are almost immediate. In GenExp it is possible to explore the genome intuitively moving it with the mouse just like geographical map applications. Additionally, in GenExp it is possible to have more than one data viewer at the same time and to save the current state of the application to revisit it later on.

**Conclusions:**

GenExp is a new interactive web-based client for DAS and addresses some of the short-comings of the existing clients. It uses client-side data rendering techniques resulting in easier genome browsing and exploration. GenExp is open source under the GPL license and it is freely available at http://gralggen.lsi.upc.edu/recerca/genexp.

## Introduction

In recent years the volume of genomic data on genome sequences and their annotations have been growing at a rapid pace. New organisms are sequenced every year, annotations on those sequences are constantly produced and refined and new data associated with these annotations is created. Most of this data is stored in public databases which are freely accessible and usually offer various options to browse and download the data, most of them via web interfaces. However, not all the databases offer programmatic access to their data nor a common format for its downloadable files. The DAS system provides a standard programmatic interface that is relatively easy to implement. This is specially important for small databases that lack the resources to develop and maintain such a system.

### The Distributed Annotation System

In an attempt to address some of these issues the Distributed Annotation System (DAS) was proposed [1], [2] (http://www.biodas.org). DAS is a client-server protocol designed to share and integrate annotations on biological sequences. It is based on standard web technologies, HTTP and XML, and offers a REST-like interface. DAS is currently widely used, with more than 1000 sources from more than 50 organizations, and some of the biggest public databases of biological data offer access to its contents via DAS [3]. The DAS Registry [4] offers a source discovery service with listings including most of the available sources. The DAS architecture was designed around the idea of having a small number of complex clients integrating data coming from many different simple sources.

DAS clients are responsible for multi-source data management, integration and representation. This means that DAS clients are usually complex applications. There are currently a number of stand-alone or web-based DAS clients available. Clients can be loosely classified into two different categories: protein-oriented clients are specialized in showing deep annotation of a relatively short sequence while genomic-oriented clients are capable of managing many annotations over a long sequence.

Protein-oriented clients usually offer a wide range of representation possibilities (3D structure, interactions graph, alignments) and most of them are stand-alone applications [5]–[9]. A notable exception is Dasty [10], a web-based protein-oriented client that creates an interactive and visually attractive representation of protein data in the browser without relying on the newest web technologies. It uses series of specially styled and positioned HTML div elements to create the graphical representation of the features and a Java applet to show the 3D structure of the protein. This approach, however, does not scale well to the data density needed in genomic-oriented clients.

### Genomic data visualization

Due to its multi-scale nature, genomic data visualization is challenging. Genomic browsers have to deal with very long sequences (in the range of hundreds of millions of bases for some chromosomes) and manage their annotations in an efficient way. Since the sizes of features vary from one base to several megabases a very wide range of zoom levels is necessary: the whole chromosome representation needs to be a million times more dense than the base-pair view while remaining informative.

Stand alone genomic browsers [11], [12] have full access to the underlying OS functionality and can take advantage of disc-based caching mechanisms and advanced drawing capabilities. On the other hand, web-based genome browsers are restricted to the web browser environment, and so, access to the underlying hardware is severely limited. No disc access is possible, memory management functions are not available and drawing is limited to HTML related technologies.

To overcome the limitations imposed by the web browser environment, most of the web-based genomic browser offload the data representation responsibilities to the server side, where static images are created to be later shown by the client running on the web browser. Ensembl [3], UCSC Genome Browser [13] and GBrowse [14] are examples of this approach. Creating the image on the server, however, usually implies a trade-off on interactivity since every change on drawing parameters (position, zoom level, active data set) results in a server request. Some attempts have been made to use tiling images, like Google Maps, but this approach has severe scalability problems given the extreme zoom range needed in genome visualization.

Client-side data rendering allows for greater interactivity, since minor changes on drawing parameters would not trigger a server request. This approach, however, has important constraints on available memory and rendering capabilities. KaryoDAS is a web-based client with client-side rendering that uses standard HTML entities to create its renderings. This imposes limitations on the number of features that can be drawn at the same time and its appearance and thus, KaryoDAS can only use DAS sources offering pre-filtering capabilities. Using newer web technologies such as HTML5 canvas it would be possible to build an interactive web-based genomic DAS client able to use any of the available DAS sources.

### Goal

The goal of this work is to take advantage of the newest web technologies (specially the canvas element on HTML5) to create a new DAS genome browser with a fully interactive user interface with real-time zooming and panning. The new browser is based on web standards and does not need any browser plugin such as Flash or Java. It is open source and freely available.

## Results and Discussion

Our genome browser GenExp -available at http://gralggen.lsi.upc.edu/genexp- offers a web-based ready-to-use interactive genome browsing experience and leverages the richness of the genomic DAS data sources with the ease of web applications.

### Overview

As a genome oriented DAS client, GenExp is able to display data coming from the available genomic DAS sources in an integrated way, creating a single representation with data from different sources represented side-by-side and precisely positioned over its common reference sequence.

Data is drawn in tracks, each one representing a certain kind of data coming from a DAS source. Groups of tracks with data from the same region viewed at the same zoom level are drawn together in a track container. Dependent and synchronized track containers can be created to show an extended region, or to view the same region at different zoom levels. It is possible, for example, to study a region at a detailed base level and at the same time keep a view of its gene and chromosome contexts.

GenExp is fully interactive and its interaction scheme has been designed to encourage the exploration of the represented data. A view can be moved by dragging it with the mouse, centered by double-clicking on it and zoomed with the mouse scroll-wheel in the same way as it is done on map-viewing applications. Detailed information on a given feature can also be shown.

While GenExp is capable of rendering whole chromosomes at once, its interactive interface excels when not making drastic changes in zoom levels, since once the data is in memory, moving around a region has no network overhead. Since drawing times are in the order of milliseconds, any delay will be due to network latency. This means that relatively small movements and zoom changes will be performed almost immediately, incurring in no noticeable delay.

Since GenExp is a web-based application, no installation is needed: it is always ready to use via the web browser. Despite its web nature, it offers a desktop-like user experience by relying on common user interaction elements and widgets, such as menus, dialogs and toolbars.

### Data Sources

A small list of sources is pre-configured in our public instance of GenExp and it is possible to add any genomic DAS source to GenExp simply entering its URL on the provided dialog. Using this feature it is possible to include even custom data by creating a new DAS source, for example using easyDAS [15] (http://www.ebi.ac.uk/panda-srv/easydas/), the automatic DAS server creation tool, and displaying it along the preexisting sources.

### Multiple Views

GenExp can manage an arbitrary number of data containers at the same time. These multiple viewers can be dependent or independent.

Dependent viewers might be either different zoom levels of the same data, in order to get a broader context view when examining a close-up view of a region for example, or “next” and “previous” regions in order to extend the region displayed at the same zoom level.

**Figure 1:**
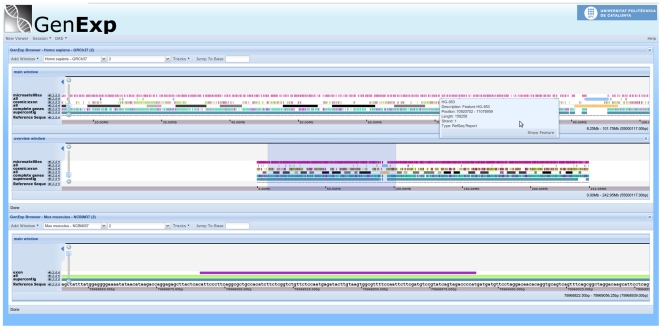
Main view of GenExp. Different regions from two organisms are represented at the same time. For human assembly GRCh37 there is a view showing more than 80 Mb and an additional linked Overview window. The other window is showing a region containing an exon at a base level view for the *Mus musculus* assembly NCBIM37.

Independent viewers can show data from any genomic region, even from other organisms, so it is possible to compare related regions of different genomes side-by-side (Figure 1).

### Sessions

Session management capabilities are included in GenExp. It is possible to save the state of the application at any given moment to later rebuild it. Session info is stored in flat text files so it is possible to store and share them.

### Customization

In addition to data customization, appearance tweaking is also in place for both the server administrator and final user. End users can customize the rendering process, selecting any of the available drawing and colouring tools or changing the individual colors used. These changes will take place instantly since no communication with the server is required. Administrators can define per-track defaults, available options, or even create new drawing routines with a few lines of code.

### Limitations

GenExp has some limitations that should be noted. First of all, due to server and network capacity, requesting large regions of very dense tracks can result in timeouts or failed requests. There are some mechanisms in DAS to prevent this but unfortunately not all servers implement them. Additionally, some incompatibilities exist with some web browsers and so GenExp has been only proven to work completely on Firefox version 1.5 or newer. The functionality available on other browsers may vary.

## Methods

### Design and Implementation

**Figure 2:**
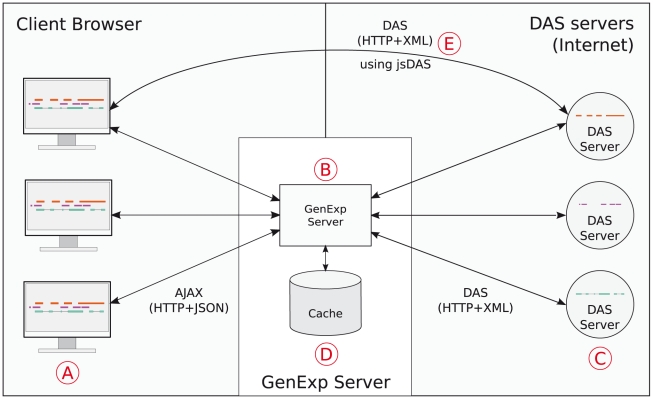
GenExp general architecture. The Javascript GenExp client (A) talks to the Perl GenExp server (B) using AJAX. The server asks the DAS servers (C) the required data, pre-processes it, stores it in the cache (D) and send it back to the clients to visualize it. When sending very lightweight queries, clients can directly connect to DAS servers using the jsDAS library (E).

While GenExp as a whole is a DAS client, the system itself has a client-server architecture with a complex web-based Javascript client talking to a simple Perl server (Figure 2). While the client is responsible for user interaction, data management and representation, the server is mainly a proxy with some basic caching capabilities.

#### The client

The client is a rich Internet application implemented in Javascript and runs inside the web browser. It is completely modular and easily modifiable and extensible.

Since GenExp creates the data representation on the client side, there is an important part of the client devoted to data gathering and management. Data is requested from the server only when needed and based on zoom level and viewing position, minimizing server load and network traffic. All received data is cached in memory so fewer requests are needed when moving and zooming.

The drawing strategy used by other genome browsers with client-side rendering is to use standard div elements to represent the genomic features. This approach has some advantages but has limitations of scalability specially of creativity, since customization possibilities are limited. GenExp, on the other hand, takes advantage of the latest HTML5 capabilities added to web browsers to create the representation of genomic features. The new canvas element exposes a low-level procedural drawing API so it is possible to create actual images on-the-fly and move them with no extra overhead. This means that when creating the representation, instead of creating a complete HTML element for every feature GenExp only draws a glyph on an image element. This has a huge impact on interactivity because the browser layout and rendering engine won't have to move hundreds or thousands of elements on every position change but only an image element.

GenExp maintains an in-memory cache on the client side. The size of this cache has not been arbitrarily limited but thanks to the feature compression scheme applied on the server side, data is only received and so stored at the required resolution. Furthermore, cache management code will maintain the cache at manageable levels by removing unused data, with a process memory footprint for typical usage between tens of megabytes and a few hundreds of megabytes.

Two Javascript libraries are used in GenExp. The Prototype library (http://www.prototypejs.org/) extends the capabilities of the basic data types and hides cross-browser issues. In particular, Prototype's custom events implementation has been used to make the application event-driven. ExtJS (http://www.extjs.com/) has been used to create and manage the graphical user interface.

The same origin policy severely limits the communications of web applications to only the server it comes from. Since GenExp needs to get data from multiple distributed sources, it sends all its requests to a proxy in its own server, which in turn fetches the data from the DAS sources.

For some lightweight DAS queries (such as getting the metadata of a newly added source) that due to their minimal size would not benefit from the the caching and compression performed by the GenExp server, the jsDAS library (http://code.google.com/p/jsdas) is used. jsDAS is a lightweight Javascript DAS client library and thanks to its Cross-Origin Resource Sharing (CORS) support, is able to connect to many DAS sources without the need of a server proxy. Since jsDAS does not implement any caching nor compression scheme, it would not be feasible to use it to retrieve feature data.

#### The server

The server subsystem is essentially a proxy and has three important additional capabilities: translation, preprocessing and caching. It has been written in Perl as a CGI script and uses the Bio::Das::Lite (http://search.cpan.org/dist/Bio-Das-Lite/) package to handle the DAS specific communication.

DAS data transport format is XML and while it is expressive, widely supported and human readable, it tends to be very verbose and somewhat clumsy to parse in Javascript. The JavaScript Object Notation (JSON) (http://json.org/), on the other hand, is simple, light and compact and native to Javascript and so is the data format used in the communication between GenExp client and server subsystems. The translation from DAS XML to GenExp JSON is performed by the server.

One of the key steps in providing zoom-dependent level of detail on the genome representation, is the server-side on-the-fly preprocessing. The server retrieves the data from the sources and creates different versions for different zoom levels, deciding on the required granularity.

The server has also caching capabilities and stores the preprocessed data for a short time, reducing the server and DAS sources load and speeding up responses. Since the caching times are short, updates on the data served by the DAS sources are propagated to the clients.

### Availability and Future Directions

GenExp is an open source project hosted at http://code.google.com/p/genexp/ and under the GPL license. An instance of GenExp is running at http://gralggen.lsi.upc.edu/recerca/genexp and can be freely accessed with no restrictions.

In the near future, it is planned to further optimize GenExp so it can reliably work with denser data sources. It will also offer more customization options support for more complex renderings. Filtering, export and analysis capabilities are also planned.
